# When more is not always better: exercise dose and children’s cognitive development from an interpretable machine learning perspective

**DOI:** 10.3389/fpsyt.2026.1908580

**Published:** 2026-08-14

**Authors:** Wenbing Zhu, Weiqiang Zhang, Liming Yong

**Affiliations:** 1 College of Physical Education and Health Science, Chongqing Normal University, Chongqing, China; 2 Department of Physical Education, Central South University, Changsha, China; 3 Institute of Physical Education, Xuzhou Kindergarten Teachers College, Xuzhou, China

**Keywords:** cognitive ability, cognitive development, exercise dose, machine learning, random forest

## Abstract

**Background:**

Exercise intervention has been associated with cognitive development in children. However, current study methods have not clarified the potential nonlinear association between exercise dosage and cognitive ability, and interpretable machine learning approaches may provide a novel perspective for exploring these complex relationships. Therefore, this study aimed to construct and verify a machine learning model of the relationship between exercise dosage and cognitive development in children.

**Methods:**

A total of 8623 valid samples from the China Family Panel Studies (CFPS) database were analyzed in this study. SPSS 25.0 was used to perform descriptive statistical analysis. SHAP values and partial dependence plots were used to enhance model interpretability. Three predictive models—Exercise Dose–Cognitive Ability Score (ED-CAS) model, Daily Physical Activity Duration–Cognitive Ability Score (DPAD-CAS) model, and Weekly Frequency of Physical Activity–Cognitive Ability Score (WFPA-CAS) model—were developed using the random forest algorithm.

**Results:**

The ED-CAS, DPAD-CAS, and WFPA-CAS models demonstrated moderate predictive performance (R² = 0.21, 0.14, and 0.11, respectively). SHAP analysis revealed mean marginal contributions of 0.12, 0.07, and 0.11 for ED, DPAD, and WFPA, respectively. Partial dependence analyses further identified normalized parameter peaks at 1.35 (ED), 1.4 (DPAD), and 0.9 (WFPA), indicating that these exercise-related features contributed differently to model predictions across dose ranges. However, dose thresholds were observed (≤720 minutes/week, ≤6 days/week, ≤85 minutes/day), beyond which CAS declined significantly.

**Conclusions:**

This study identified a potential inverted U-shaped association between exercise dosage and cognitive ability in children. The observed dose ranges may provide preliminary reference information for future exercise intervention. However, longitudinal and experimental studies are required to determine causal relationships and establish evidence-based exercise recommendations.

## Introduction

### Cognitive abilities: an important ability for children’s development

Cognitive ability refers to the human brain’s ability to process information. It reflects the brain’s efficiency in information processing, environmental adaptation, and problem solving (1). Key dimensions include attention, the ability to focus on specific tasks or stimuli; memory, encoding, storage, and retrieval of information; language, comprehension, expression, and application of linguistic systems; thinking, analysis, synthesis, and evaluation of information; perceptual ability, recognition and interpretation of sensory stimuli (visual, auditory, tactile); learning, acquisition of new knowledge and skills; reasoning, deriving conclusions from existing data; and decision-making, strategic selection among alternatives (2, 3).

Childhood is a critical stage in life for the development of cognitive abilities, which can be associated with many aspects of a child’s development (4). Studies have revealed an important link between cognitive ability and academic performance. For example, children who pay attention tend to score higher on math and reading comprehension tests (5–7). Whereas efficient memory encoding enhances children’s ability to understand and apply complex concepts, perceptual skills lay the cornerstone for early math symbol comprehension and geometry learning (8, 9). Meanwhile, cognitive ability has a profound impact on children’s social adaptation (10), language ability on children’s peer interactions (11), reasoning ability on children’s understanding of social norms and equity (12), and decision-making capacities on children’s strategic choices in social conflict (13). In addition, cognitive ability is associated with motor coordination, creativity, and goal-directed behavior (14, 15). Therefore, examining factors associated with children’s cognitive abilities is essential to promote their overall cognitive health and physical development.

In recent years, some studies on the relationship between children’s cognitive improvement and physical activity have gradually entered the societal view and have become a topic of focus in interdisciplinary fields such as exercise science and neuroscience (16–19). A recent study found that moderate, regular exercise not only has a positive impact on children’s cognitive development but also improves their academic performance (16). Another study has also observed that exercise can accelerate cognitive processing and help resolve conflicts related to visual-spatial stimuli (17). Additionally, other studies have pointed out that physical activity can improve the allocation of attention resources (18) and optimize memory encoding mechanisms to enhance memory retention (19). Previous literature suggests that the benefits of physical activity on cognitive development may involve exercise-related changes in brain function, including neuroplasticity, cerebral blood flow regulation, and neurotransmitter modulation (20). This process includes changes at the cellular and molecular levels, adjustments in how the brain is structured and works, improved neuroplasticity, and more neurotransmitter release—these factors all work together to contribute to the prediction cognitive development (21).

However, the sample sizes of existing studies are relatively small, and the research methodology is more focused on exploring the relationship between physical activity and cognitive performance. At the same time, there is a lack of research on how specific dosage parameters (frequency, intensity, and duration) modulate cognitive performance. Furthermore, conventional linear models fail to capture “optimal zones accurately” and the threshold effects of exercise dosage due to inherent heterogeneity among children (22). Consequently, developing novel modeling approaches to quantify dose-cognition relationships represents an urgent research topic.

### Machine learning: new breakthroughs

Machine learning (ML), a subfield of artificial intelligence, autonomously identifies and refines patterns in data through experiential learning (16) —the applications of ML span diverse domains (16, 23). In sports injury prevention, Karnuta et al. (2020) employed ML to predict injury risks for 13,982 baseball players in the upcoming season, achieving 70% prediction accuracy (23). Similarly, Kunze et al. (2020) utilized ML to analyze wearable device data from patients undergoing total knee arthroplasty, modeling multidimensional metrics to correlate higher postoperative functional outcomes with improved clinical recovery (24).

In mental health, Lamb et al. (2024) integrated neurocognitive data with ML algorithms to enhance the precision and reliability of predictive models (25). Yan et al.(2023) used ML to identify key risk factors for bullying among Chinese adolescents, pointing to the most critical predictors outside of these domains (26). ML is uniquely suited for processing high-dimensional nonlinear data, detecting complex interactions, and enabling personalized predictions, which has driven its application in healthcare, education, and public services. These capabilities also provide new methodological avenues for studying the dynamic relationship between exercise dose and cognitive performance (16).

This study addresses the above issues by using the random forest model to explore nonlinear associations between exercise dose and children’s cognitive ability, identify predictive patterns across different exercise dose ranges, and examine whether the observed associations are consistent with an inverted-U pattern (27).

## Methods

### Data sources

The data for this study were derived from the China Family Panel Studies (CFPS). The CFPS is a nationwide longitudinal survey organized by the Institute of Social Science Surveys (ISSS) at Peking University. The survey covers multidimensional assessments of communities, families, and individuals, involving multiple research topics (28, 29). Among them, the 2013–2014 survey involved exercise-related indicators, which provided a reliable data source for this study. The CFPS database contains 9449 survey reports on adolescents aged 14–15. After data cleaning, 728 individuals with incomplete data were excluded, and 148 individuals with outliers were identified and excluded using the Z-score method. Finally, 8623 individuals were included in the analysis.

### Variable definitions

The main variables of the CFPS included daily physical activity duration (DPAD), weekly frequency of physical activity (WFPA), and standardized cognitive ability score (CAS). Additionally, we constructed an integrated, exercise dose indicator (ED), using DPAD and WFPA(ED = DPAD × WFPA). Because exercise exposure includes multiple dimensions, separate models were developed for ED, DPAD, and WFPA to examine whether cumulative volume or individual dose components showed different predictive pattern.

### Statistical analysis

SPSS 25.0 was used to perform descriptive statistical analysis (30), PyCharm 2024 for machine learning model development, and the visualization tools SHAP (31) (Shapley Additive exPlanations) plots and Partial Dependency Plots (32) (PDPs) were used to improve the interpretability of the models. Association between DPAD and CAS, WFPA and CAS, and ED and CAS was explored using correlation analysis, controlling for gender and age variables, respectively.

To further validate the nonlinear associations identified by machine learning models, generalized additive models (GAMs) with penalized spline functions were conducted. Restricted cubic spline (RCS) regression models were additionally applied to examine potential nonlinear dose-response patterns between exercise dose indicators (ED, DPAD, and WFPA) and CAS. The number and location of knots were determined based on Akaike information criteria and distribution characteristics of exercise variables.

### Model construction

The model was constructed using the random forest regression algorithm. Random Forest is a type of ensemble learning method in machine learning. It improves the accuracy and generalization ability of the model by constructing multiple random decision trees. Each decision tree is independently constructed based on randomly sampled data and features. The final prediction results are integrated through voting (for classification) or averaging (for regression). It is suitable for modeling classification or regression tasks (33). This study’s random forest comprised B = 200 decision trees generated through recursive binary splits using the Gini impurity minimization criterion for node splitting (34). Where p(c|t) denotes the proportion of samples belonging to class *c* at node *t*.


$$\text{Gini}(t)=1-\sum_{c=1}^{C}\;p{\left(c|t\right)}^{2}$$

The grid search method was employed to identify the optimal parameter combination (34) was employed to identify the optimal parameter combination. The optimization of each hyperparameter aimed to maximize the information captured from the training data, thereby achieving a more precise model fit between the training and testing datasets. The number of trees (n_estimators) was set to {100, 200, 300}, the maximum depth (max_depth) to {5, 10, 15}, and the minimum number of samples per leaf (min_samples_leaf) to {1, 5, 10}.

### Model validation and interpretation

Model validation employed a 10-fold cross-validation approach (33). The dataset was randomly partitioned into 10 equal subsets (folds). One-fold served as the test set for each iteration, while the remaining nine were used for training. This process was repeated 10 times, with each fold acting as the test set once. The cross-validation was repeated three times to mitigate random variability. Model performance was evaluated using Mean Squared Error (MSE) and the coefficient of determination (R^2^):


$$MSE=\frac{1}{n}\sum_{i=1}^{n}{\left(y_{i}-{\hat{y}}_{i}\right)}^{2}$$

$$R^{2}=1-\frac{\sum {\left(y_{i}-{\hat{y}}_{i}\right)}^{2}}{\sum {\left(y_{i}-\bar{y}\right)}^{2}}$$

The interpretability of the model was achieved through SHAP values (31) and PDP (32). SHAP uses Shapley values to measure the contribution of each feature to the model prediction, while Shapley values ensure the fair distribution of cooperative gains, accurately reflecting the influence of each feature. SHAP plots provide a global view of feature importance and local insights into individual predictions. They intuitively display the relationship between feature values and predictions through color and position, thereby enhancing interpretability. PDP reveals the relationship between the target variable and features—whether it is linear, monotonic, or more complex—thereby aiding in understanding and identifying the influence of each feature in the random forest model. SHAP values use cooperative game theory to quantify feature contributions:


$$\phi_{j}=\sum_{S\subseteq F\setminus \{j\}}\frac{|S|!(|F|-|S|-1)!}{|F|!}[f(S\cup \{j\})-f(S)]$$

where F is the full feature set, S is a subset of features, and f is the model prediction function.


$${\hat{f}}_{S}(x_{S})=\frac{1}{n}\sum_{i=1}^{n}\hat{f}(x_{S},x_{C}^{\left(i\right)})$$

where $x_{S}$ is the feature of interest, $x_{C}^{\left(i\right)}$ is the actual value of the other features in the dataset, and n is the size of the dataset.

## Results

### Descriptive statistics

Descriptive statistics for all key variables are presented in **Table 1**. The sample consisted of 8623 participants. The mean score for daily physical activity duration (DPAD) was 41.22 minutes (SD = 27.59), with scores ranging from 0 to 180 minutes. The weekly frequency of physical activity (WFPA) averaged 3.36 times per week (SD = 1.92), within a possible range of 0 to 7. The average eED score was 138.24 (SD = 118.23), with considerable variation (range: 0–780). Cognitive adjustment score (CAS) ranged from –2.18 to 2.06, with a mean of 0.35 (SD = 0.79). Demographic variables (age and gender) were recorded for all participants but not analyzed in the current section.

**Table 1:** Descriptive statistical results.

Variable	N	Min	Max	M	SD
Age	8623				
Gender	8623				
DPAD	8623	0	180	41.22	27.59
WFPA	8623	0	7	3.36	1.92
ED	8623	0	780	138.24	118.23
CAS	8623	-2.18	2.06	0.35	0.79

### Correlation analysis

Pearson correlation coefficients among DPAD, WFPA, ED, and CAS are reported in **Table 2**. The results showed a strong positive relationship between DPAD and ED (*r* = 0.605, *p* <.001), and also between WFPA and ED (*r* = 0.692, *p* <.001), meaning that more physical activity was linked to higher ED scores in this group. Both DPAD and WFPA also showed weak but statistically significant positive correlations with CAS (*r* = 0.047 and *r* = 0.171, respectively; both *p* <.001). Similarly, ED was weakly positively correlated with CAS (*r* = 0.135, *p* <.001). While statistically significant, the effect sizes for associations involving CAS were small, indicating limited practical significance.

**Table 2:** Correlation analysis.

Predictive models	DPAD	WFPA	ED	CAS
DPAD	1			
WFPA	0.055**	1		
ED	0.605**	0.692**	1	
CAS	0.047**	0.171**	0.135**	1

**Correlation is significant at the 0.001 level (two-tailed).

### Model performance

For the ED-CAS model, the MSE was 0.59 (95% CI: 0.55-0.63), and the R² was 0.21 (*p* < 0.001). For the WFPA-CAS model, the MSE was 0.57 (95% CI: 0.55-0.63), and the R² was 0.11 (*p* < 0.001). For the DPAD-CAS model, the MSE was 0.60 (95% CI: 0.55-0.63), and the R² was 0.14 (*p* < 0.001). Although statistically significant, the explained variance was moderate, suggesting that exercise-related variables represent only one component among multiple determinants influencing children’s cognitive ability.

### Nonlinear association analysis

GAM and restricted cubic spline analyses further supported the nonlinear associations between exercise dose indicators and cognitive ability. The spline curves demonstrated increasing predicted CAS values at lower-to-moderate exercise dose ranges, followed by a plateau or decreasing trend at higher dose levels, showing patterns consistent with the PDP results from random forest models.

### Feature importance analysis

**Figure 1:**
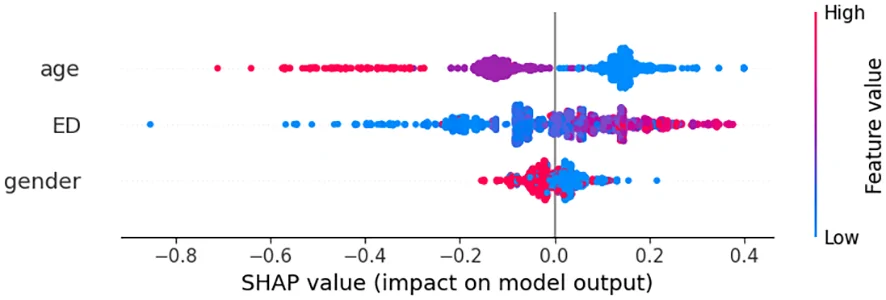
SHAP of ED-CAS.

**Figure 2:**
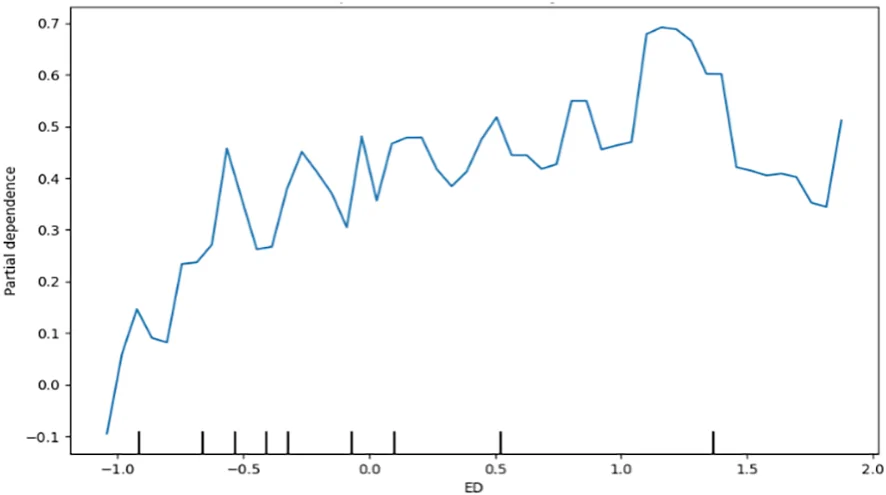
PDP of ED-CAS.

**Figure 3:**
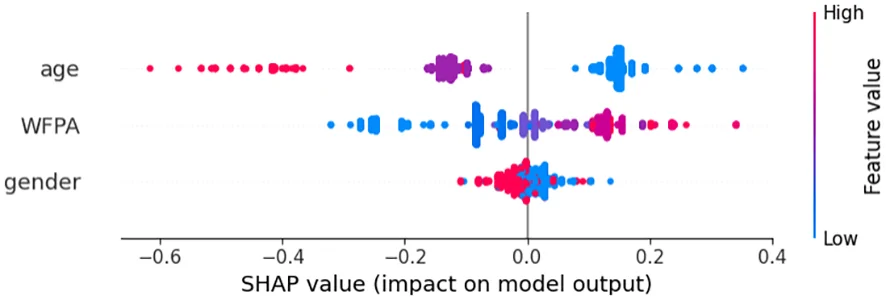
SHAP of WFPA-CAS.

**Figure 4:**
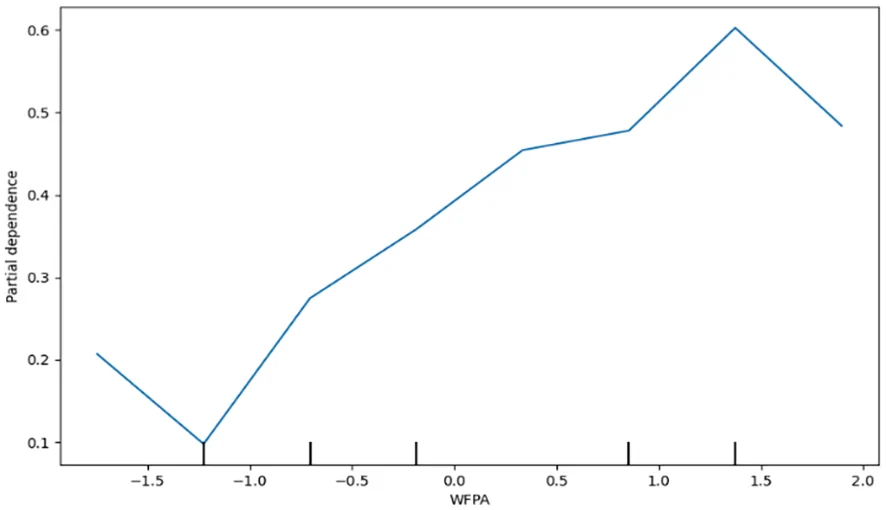
PDP of WFPA-CAS.

**Figure 5:**
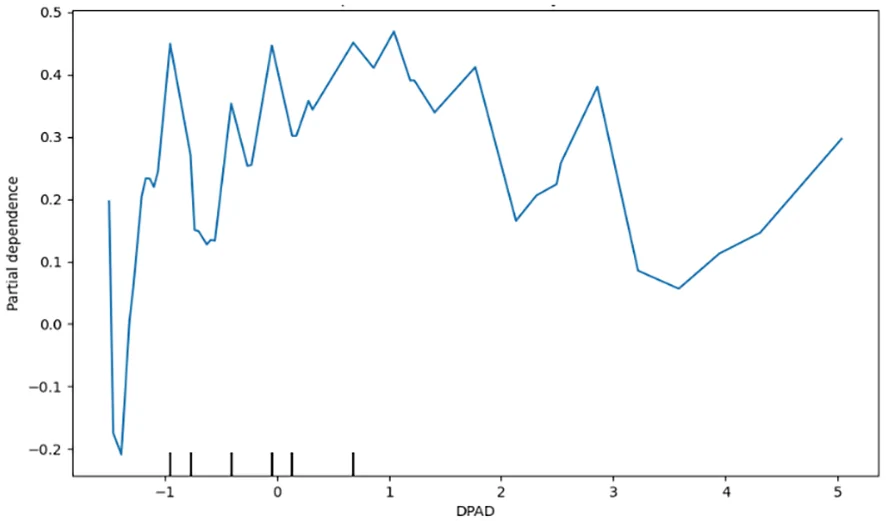
PDP of DPAD-CAS.

**Figure 6:**
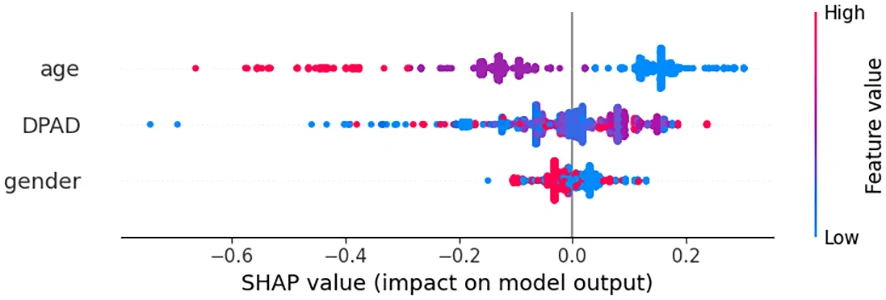
SHAP of DPAD-CAS.

The feature contribution analysis based on SHAP values revealed an average marginal contribution of 0.12 for ED, indicating its relative contribution to model prediction rather than a direct effect on cognitive ability. With its SHAP plot displayed in **Figure 1** and a standardized peak parameter of 1.35 in the PDP shown in **Figure 2**. WFPA had an average marginal contribution of 0.11, illustrated in **Figure 3**, and a standardized peak parameter of 1.4 in the PDP in **Figure 4**. DPAD’s average marginal contribution was 0.07, depicted in **Figure 5**, with a standardized peak parameter of 0.9 in the PDP in **Figure 6**.

## Discussion

This study used a random forest model to illustrate the nonlinear relationship between exercise dose and children’s cognitive ability. ED, WFPA, and DPAD showed positive predictive associations with CAS within specific observed ranges. The model identified potential turning points (≤720 minutes/week, ≤6 days/week, ≤85 minutes/day), beyond which predicted CAS values showed decreasing trends. The results reinforce the view that an appropriate level of exercise can enhance children’s cognitive functioning, in line with prior evidence. For example, Erickson et al. (2011) study had children perform aerobic exercise. Results showed that doing this four times a week can significantly improve children’s working memory (35). Chen et al. (2016) study used acute moderate-intensity aerobic exercise lasting 30 minutes and found that children’s working memory was enhanced (36). However, these two studies raise new questions about the impact of exercise type and frequency on children’s cognitive abilities, which is worth further research. In a longitudinal study, researchers recorded children’s physical activity at age 13 and their academic performance at age 16. The analysis showed that children’s academic performance at 16 is related to their physical activity at 13 (37). A meta-analysis of 24 reports shows a beneficial link between children’s exercise and later cognitive and academic performance (38), indicating that exercise may continuously benefit children’s cognitive ability. This suggests exercise may have sustained cognitive benefits for children. This aligns with the neurometabolic “cumulative effect hypothesis” (39), where total exercise volume predominantly drives cognitive gains. Sustained stimulation is needed for hippocampal neurogenesis (40). Additionally, high-frequency exercise promotes the continuous release of neurotrophic factors (41), which may contribute to cognitive development through neurobiological pathways (42).

The present analysis further clarifies the nonlinear, inverted U-shaped relationship between exercise dosage and children’s cognitive abilities. When exercise dosage exceeds the observed range associated with higher predicted cognitive scores, the predictive pattern showed a downward trend, although whether excessive exercise directly impairs cognition requires further investigation. This result aligns with Mehren et al. (2019), who found that moderate-intensity aerobic exercise benefits children’s cognition more than high-intensity exercise (43). Similarly, a meta-analysis indicates that exercise interventions lasting over 30 minutes per session improve children’s executive function more effectively than shorter ones (44). Therefore, this finding suggests that an optimal exercise dosage range exists. Low exercise volumes may not stimulate the hippocampus, while excessive exercise might increase cortisol levels, induce oxidative stress, and may indicate hippocampal neuron apoptosis, hindering cognitive development (45). This exercise-induced molecular mechanism complements our findings.

To further clarify dose-specific contributions, SHAP value analysis was applied to examine the relative contribution of different exercise dose indicators to model predictions. It also showed that the gain efficiency from different exercise dosage dimensions varies within a specific range; a one-unit increase in weekly exercise time (e.g., 60 minutes) boosts CAS by 12%, a more significant effect than daily exercise duration (7%) and weekly frequency (11%). This further confirms the hypothesis that exercise dosage has a cumulative effect on children’s cognitive abilities. Moreover, the threshold for weekly exercise frequency (≤6 days/week) emphasizes the importance of at least one full rest day to promote neural recovery, which is consistent with the synaptic homeostasis plasticity (SHY) theory (46).

Despite these insights, several limitations warrant attention. While the random forest model helped capture complex nonlinear patterns, its predictive power was constrained by reliance on a single data source. The modest R² value also demonstrates that exercise-related variables alone cannot fully explain individual differences in cognitive ability, which is influenced by multiple biological, psychological, educational, and environmental factors (such as neurotrophic factor concentration and cerebral blood flow dynamics) and environmental factors (such as family and exercise environment).

Nevertheless, this study provides empirical support for child health policies, including school curriculum reforms, community health programs, and interventions for children with special needs. Future research in sports health should focus on multimodal data fusion to build an “exercise-brain-behavior” model, quantifying the neural mechanisms’ contributions to dose-cognition relationships. Additionally, developing real-time feedback systems based on reinforcement learning could dynamically adjust exercise prescriptions according to children’s cognitive states (e.g., classroom attention scores), better promoting cognitive development.

## Conclusion

This study applied an interpretable random forest approach to explore nonlinear associations between exercise dose indicators and cognitive ability among children. The findings suggest that ED, WFPA, and DPAD contain predictive information regarding cognitive ability, with potential nonlinear patterns observed across different exercise dose ranges. However, the identified turning points should not be interpreted as causal thresholds or definitive optimal exercise prescriptions. Future longitudinal and intervention studies are needed to determine whether these associations represent causal effects and to establish evidence-based exercise recommendations.

## Data Availability

Publicly available datasets were analyzed in this study. This data can be found here: https://cfpsdata.pku.edu.cn.
